# Professional Perspectives and Research Challenges Among AO CMF Surgeons in the Middle East and North Africa

**DOI:** 10.3390/cmtr19010005

**Published:** 2026-01-19

**Authors:** Khalid Abdel-Galil, Ammar Khalafalla, Mohamed Amir

**Affiliations:** 1 Sheikh Tahnoon Bin Mohamed Medical City, Tawam Hospital, Abu Dhabi P.O. Box 15258, United Arab Emirates; 2 Hamad Medical Corporation, Doha P.O. Box 3050, Qatar; amarkhider15@gmail.com (A.K.); drmohdamir94@gmail.com (M.A.)

**Keywords:** craniomaxillofacial surgery, research barriers, Middle East, North Africa, academic productivity, AO PEER

## Abstract

Purpose: Research drives clinical advancement in oral and craniomaxillofacial surgery by generating evidence that guides practice and innovation. However, limited literature exists describing research engagement among surgeons within AO CMF in the Middle East and North Africa. This study evaluated awareness, participation, and perceived barriers to research among AO CMF members and affiliated surgeons in the MENA region. Methods: A cross-sectional, questionnaire-based survey was distributed electronically to AO CMF members, affiliates, and professional CMF surgeon networks between October and December 2024. The 14-item survey assessed demographics, research awareness, attitudes, productivity, and barriers. Responses were anonymized and analyzed descriptively using SurveyPlanet analytics. Results: A total of 144 surgeons from 21 countries completed the survey. Pakistan (35%), Morocco (9.8%), Kuwait (7.7%), and the United Arab Emirates (7%) contributed the largest proportions. Most respondents (47.6%) expressed strong interest in research but reported difficulty initiating projects, while 32.2% cited lack of time as a major constraint. The most frequently reported barriers included challenges in research methodology (14.6%), publishing (14.6%), and manuscript writing (14.1%). Only 18.9% of participants had published more than ten articles, while 29.4% had none. Mentorship demand was high (94.4%), but awareness of the AO PEER program remained limited (37.8%). Conclusion: Surgeons expressed strong enthusiasm for research yet face substantial barriers. Strengthening research methodology training, establishing structured mentorship, expanding AO PEER engagement, and facilitating multicenter collaboration are key strategies to enhance research productivity across the region.

## 1. Introduction

Research is fundamental in advancing evidence-based practice in craniomaxillofacial (CMF) surgery. Globally, disparities in academic productivity stem from challenges such as limited institutional support, insufficient methodological training, and time constraints [1,2,3,4,5]. Similar patterns have been reported in low- and middle-income settings, where limited infrastructure and mentorship hinder research output [5,6,7].

In the Middle East and North Africa (MENA), the CMF research infrastructure is evolving, with significant variability in academic resources, funding, and research expectations across countries. Prior regional surveys conducted among AO CMF surgeons in Asia-Pacific [5] and Latin America [6] demonstrated strong interest in research but highlighted persistent barriers in methodological support, mentorship, and time availability.

Despite this, no study has comprehensively evaluated research awareness, engagement, and barriers among AO CMF surgeons working within the MENA region. This study aims to fill this gap by evaluating research participation, awareness, and perceived obstacles among surgeons affiliated with AO CMF.

## 2. Materials and Methods

### 2.1. Study Design and Participants

A cross-sectional survey was conducted between October and December 2024 among CMF surgeons and trainees affiliated with AO CMF or active in regional CMF clinical practice.

Inclusion criteria included practicing CMF surgeons or trainees based in MENA with AO CMF membership/affiliation or participating in AO CMF educational exchanges.

Exclusion criteria included responses from non-surgeons, duplicated responses, or incomplete surveys.

### 2.2. Survey Distribution

Although the primary target population was AO CMF members and affiliates, the survey link was also disseminated through professional surgeon networks, WhatsApp groups, and regional CMF communities. This resulted in participation from both members and non-members, allowing for a more comprehensive view of regional research engagement.

### 2.3. Survey Instrument

A structured 14-item questionnaire collected data on demographics, research awareness, attitudes, productivity, and barriers. The questionnaire was piloted for clarity prior to distribution.

### 2.4. Data Collection and Analysis

Responses were collected anonymously online via SurveyPlanet.com. Descriptive statistics were generated using SurveyPlanet analytics. No further analytical statistical tests were undertaken. Ethical approval was not required for this survey-based qualitative research, and informed consent was implied through voluntary participation.

## 3. Results

### 3.1. Demographics

**Figure 1:**
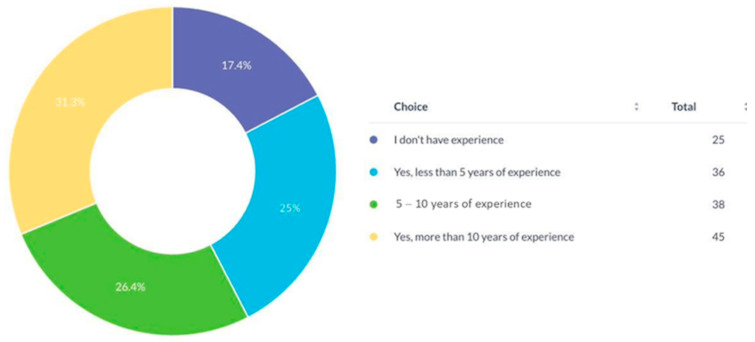
Years of experience of respondents in CMF surgery.

Of 144 respondents from 21 countries, most were from Pakistan (35%), Morocco (9.8%), Kuwait (7.7%), and the UAE (7%). About 31.3% had over 10 years of surgical experience, while 42.4% were early-career surgeons (Figure 1).

### 3.2. Research Interest and Involvement

**Figure 2:**
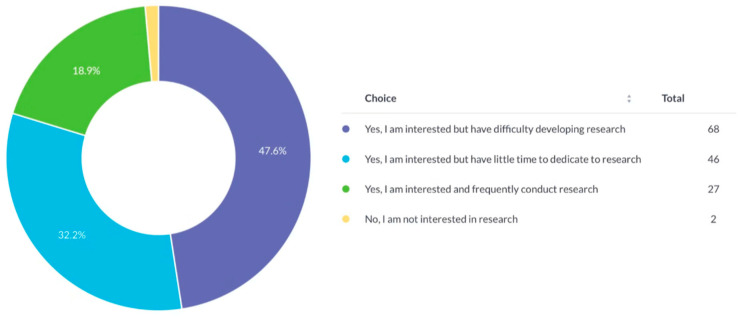
Interest of respondents in conducting research.

Nearly half of respondents (47.6%) expressed strong interest in research but faced difficulties initiating projects. Another 32.2% reported limited available time. Only 18.9% regularly engaged in research, and 1.4% had no interest (Figure 2).

### 3.3. Barriers to Research

**Figure 3:**
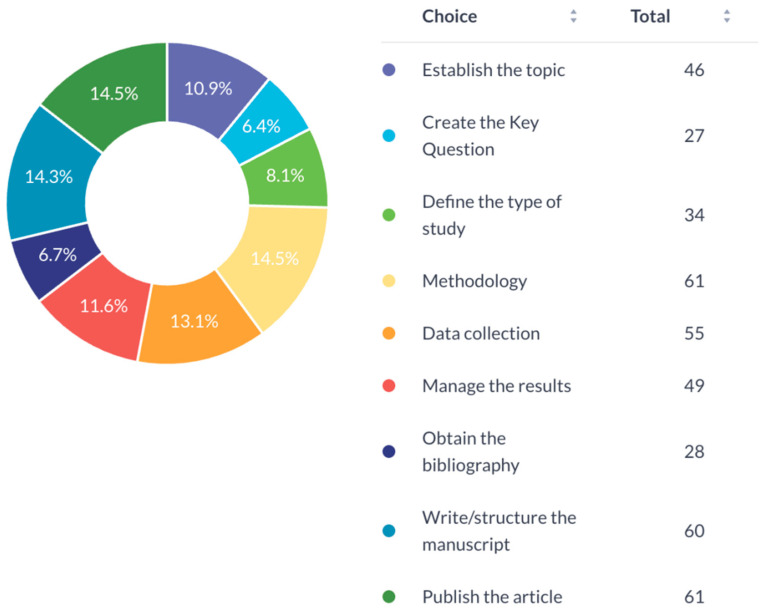
Main difficulties faced in conducting research.

**Figure 4:**
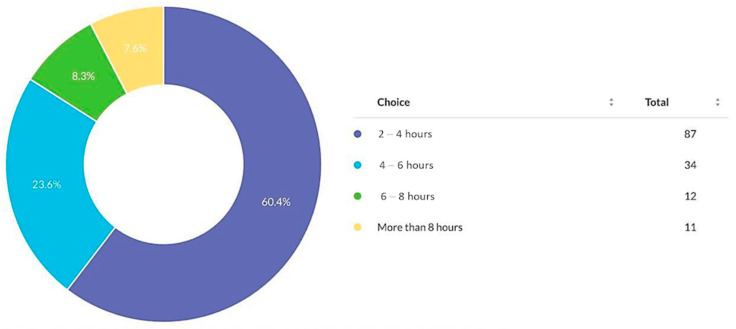
Weekly availability of respondents to dedicate time to research.

**Figure 5:**
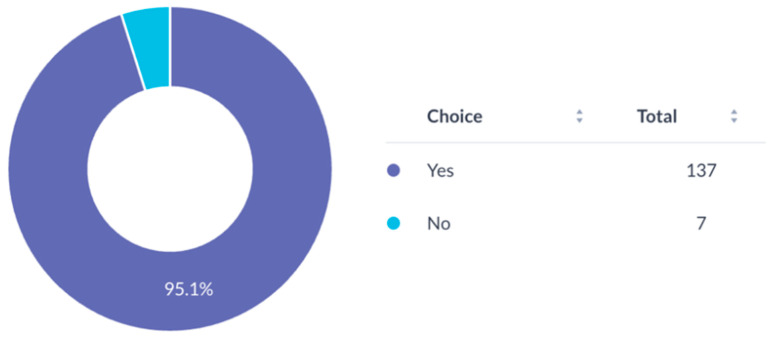
Interest of respondents in writing scientific research.

Common obstacles included challenges in research methodology (14.5%), publishing (14.5%), manuscript writing (14.3%), and data collection (13.1%) (Figure 3). Most surgeons (60.4%) could allocate only 2–4 h weekly to research (Figure 4). In addition, most respondents expressed interest in writing scientific research articles, despite reported barriers. (Figure 5)

### 3.4. Research Productivity

**Figure 6:**
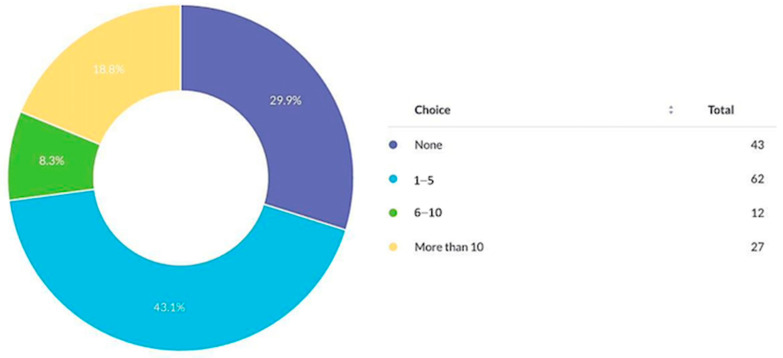
Number of scientific articles published by respondents.

A total of 29.9% had no publications, 43.1% had 1–5 articles, 8.3% had 6–10, and 18.8% had more than 10 publications (Figure 6).

### 3.5. Mentorship and Training

**Figure 7:**
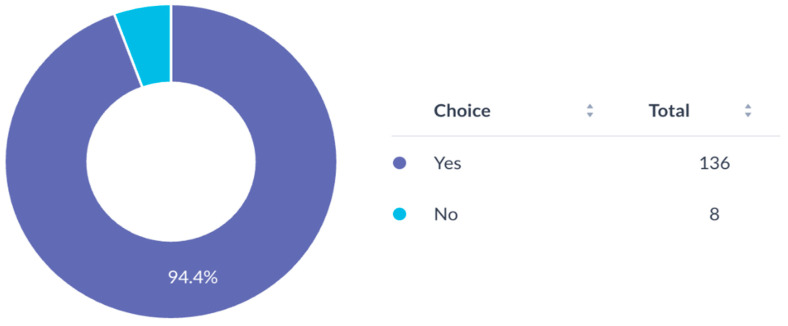
Interest of respondents in obtaining mentorship for research project development.

**Figure 8:**
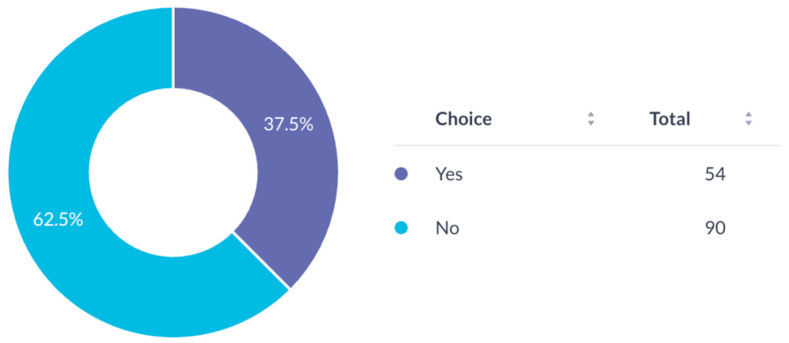
Familiarity of respondents with AO PEER program.

**Figure 9:**
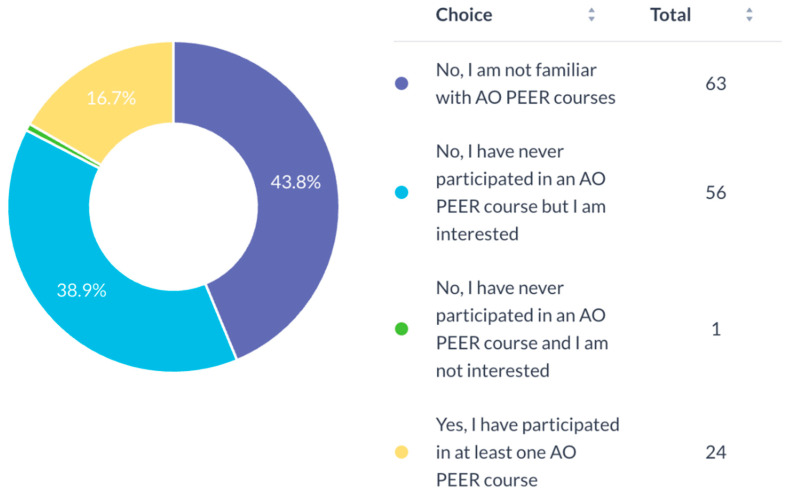
Participation of respondents in AO PEER courses.

**Figure 10:**
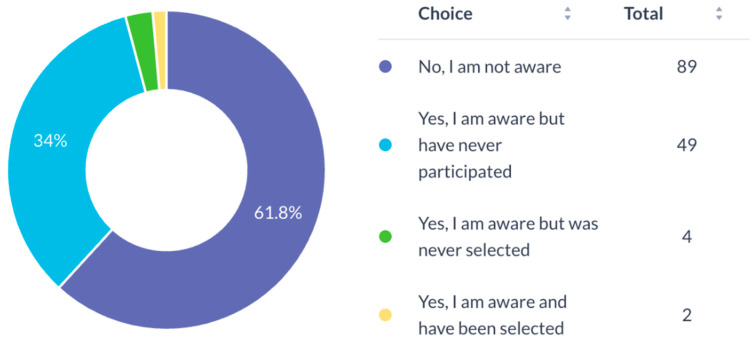
Awareness of respondents regarding AO Foundation research grant opportunities.

Most participants (94.4%) expressed a need for mentorship for research development (Figure 7). Awareness of the AO Program for Education and Excellence in Research (AO PEER) was low (37.5%) (Figure 8), and only 16.7% had participated in AO PEER courses. However, 38.9% expressed strong interest in joining such programs (Figure 9). Awareness of AO Foundation research grant opportunities was also limited among respondents (Figure 10).

### 3.6. Collaboration and Membership

**Figure 11:**
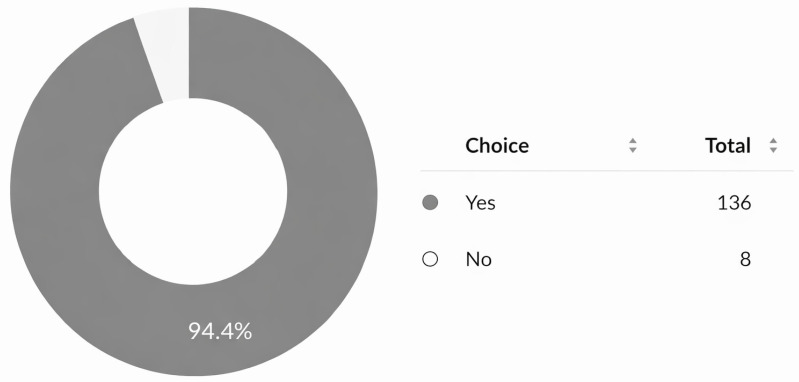
Interest of respondents in participating in multicenter research with AO CMF MENA.

**Figure 12:**
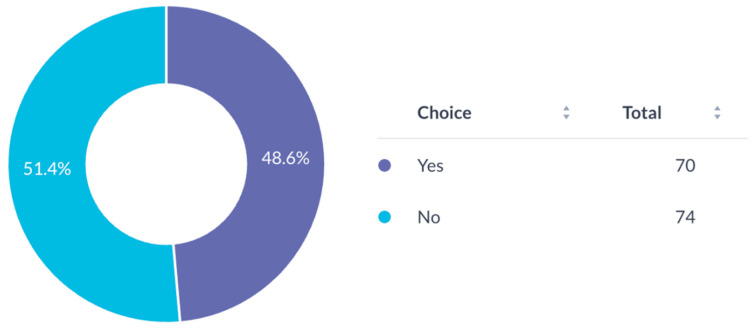
AO membership status of respondents.

Nearly all respondents (94.4%) were interested in multicenter collaborations via AO CMF (Figure 11). AO membership was evenly distributed amongst respondents (48.6% members vs. 51.4% non-members) (Figure 12).

## 4. Discussion

This study demonstrates strong research interest among CMF surgeons across the MENA region. However, this interest does not consistently translate into scholarly output, largely due to methodological difficulties, limited protected time, and lack of structured mentorship. The proportion of respondents expressing interest mirrors findings from Asia-Pacific (99%) and Latin America (96%) studies within AO networks [6,7]. However, the proportion actively engaged in research remains lower in MENA, underscoring challenges in project development and time management.

Barriers such as methodological uncertainty, lack of writing experience, and limited publication support have been reported globally [1,2,3,4,5]. These findings emphasize the need for structured mentorship and institutional programs to enhance research literacy. Earlier exposure and training in principles of research methodology was found to be linked to a more positive attitude towards scientific methodology [8].

The limited time available for research—typically under four hours per week—reflects heavy clinical demands, consistent with global data [1,2,3,4,5]. Addressing this requires systemic support, such as protected academic time and institutional incentives.

Mentorship and training through programs like AO PEER could address many reported challenges. Increased participation in such initiatives, alongside collaborative multicenter studies, could help close the productivity gap between MENA and other regions.

Initiatives such as the current AO CMF pilot research mentorship program in Latin America are eagerly awaited and will be analyzed, and potential for replication globally may be considered for wider benefit.

Similarly, an important initiative for online research collaboration, the AO CMF MENA Research forum [https://apps.apple.com/gb/app/myao-2-0/id6749233945, accessed on 15 January 2026], has been setup to share ideas, protocols, and mentorship opportunities. This forum currently has 190 active surgeon members across the MENA region.

### Comparison with Similar Recent Surveys in Latin America and Asia-Pacific

The findings of the present study align with recent region-specific investigations conducted under the AO CMF global network. Menon et al. examined research awareness and engagement among craniomaxillofacial surgeons in the Asia-Pacific region [6], while dos Santos Pereira et al. conducted a comparable analysis in Latin America [7].

In the Asia-Pacific study, nearly all respondents (99.4%) expressed interest in research, and 80% reported previous participation in academic projects. Despite this enthusiasm, limited time availability and difficulties in data management, publishing, and topic development were the main barriers identified [6]. The Latin American cohort reported a similarly high interest rate (96%), but participants demonstrated a comparatively greater publication record—over 60% had authored more than five articles [7].

When compared with these regions, the MENA group in the present study demonstrated equivalent motivation but markedly lower publication productivity, with nearly 30% reporting no publications. Barriers such as limited methodological training and insufficient mentorship were also more prevalent in MENA than in the Asia-Pacific and Latin American studies. These discrepancies may reflect differences in institutional research support, mentorship structures, and access to continuing education initiatives such as AO PEER courses.

Collectively, the three studies underscore that, while enthusiasm for research among CMF surgeons is universal, the translation of this interest into a tangible scholarly output depends strongly on regional support systems. Strengthening mentorship, providing structured research education, and facilitating cross-regional multicenter collaborations could therefore help harmonize global research productivity within the AO CMF community.

## 5. Limitations

This study has limitations, including the use of convenience sampling through AO mailing lists and informal professional networks, which may not capture the entire CMF surgeon population, and reliance on self-reported data that is subject to recall and response bias, as well as unequal geographic representation, with some MENA countries contributing very few participants.

## 6. Conclusions

Craniomaxillofacial surgeons in the MENA region exhibit strong motivation to engage in research but face persistent barriers, primarily related to time, mentorship, and methodological knowledge. Efforts to expand mentorship networks, integrate AO PEER educational programs, and support multicenter collaborations are vital in fostering a more robust research culture across the region.

## Data Availability

Data supporting the reported results are available from the authors.
